# Treatment strategy and endpoint of catheter ablation for bi‐atrial tachycardia after substrate modification ablation in a low voltage zone of the left atrial anterior wall: Long‐term results

**DOI:** 10.1002/joa3.12558

**Published:** 2021-05-24

**Authors:** Tomoyuki Arai, Rintaro Hojo, Sayuri Tokioka, Takeshi Kitamura, Seiji Fukamizu

**Affiliations:** ^1^ Department of Cardiology Tokyo Metropolitan Hiroo Hospital Tokyo Japan

**Keywords:** Bachmann's bundle, bi‐atrial tachycardia, catheter ablation, mitral isthmus, tachyarrhythmia

## Abstract

**Background:**

The termination of bi‐atrial tachycardia (BiAT) via the ablation of the Bachmann's bundle (BB) and mitral isthmus (MI) has been previously reported; however, the strategy and long‐term results of catheter ablation for BiAT remain unclear.

**Methods:**

The data of nine patients with BiAT who underwent low voltage zone (LVZ) ablation of the left atrial anterior wall (LAAW) after pulmonary vein isolation were reviewed. Patients with a P wave duration <100 ms during sinus rhythm underwent MI ablation and those with a P wave duration >100 ms underwent BB ablation.

**Results:**

MI ablation was performed in three patients and six patients underwent BB ablation. The difference in the P wave duration before and after ablation was significantly different between the ablation sites (MI group: 5.0 ms difference; BB group; 38.5 ms difference; *P* = .024). The P wave duration was prolonged by >20 ms and was 120 ms or more after ablation in 5/6 patients who underwent BB ablation. The total recurrence rate was 11.0% (mean: 26.9 months).

**Conclusion:**

The recurrence of BiAT after MI or BB ablation is low. When BB ablation was performed, the P wave duration was prolonged by >20 ms and was at least 120 ms after the ablation, which may be an endpoint that can be used to measure the success of the ablation.

## Graphical abstract

We selected the Bachmann's bundle or mitral isthmus as the treatment site for BiAT according to the patients’ P wave duration. When Bachmann's bundle ablation for BiAT was selected, the P wave duration was prolonged by >20 ms and was ≥120 ms after the ablation, which might be the endpoint of successful treatment.

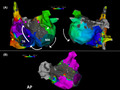

## 1 INTRODUCTION

Catheter ablation, including pulmonary vein isolation (PVI), is a generally accepted therapy for atrial fibrillation (AF). However, recurrent AF and left atrial tachycardia (AT) are likely to occur after PVI.^1^, ^2^ Several studies have reported that additional substrate modifications after PVI, such as anatomical line and low voltage zone (LVZ) ablations of the left atrial anterior wall (LAAW), reduce AF recurrence.^3^ However, a previous study revealed that LAAW substrate modification leads to bi‐atrial tachycardia (BiAT),^4^ a rare form of atrial macro reentrant tachycardia in which the reentry circuit is located in both the right and left atria (RA and LA, respectively).^5^ Several reports have indicated that BiAT can be terminated via the ablation of the Bachmann's bundle (BB) and anatomical linear ablation, especially mitral isthmus (MI) ablation.^6^, ^7^, ^8^ However, the treatment strategy and long‐term results of catheter ablation for BiAT remain unclear. This study aimed to investigate the optimal ablation sites to treat BiAT termination and the long‐term results of catheter ablation for BiAT.

## 2 METHODS

### 2.1 Study population

A total of 265 patients who underwent PVI from April 2017 to April 2019 at Tokyo Metropolitan Hiroo Hospital were enrolled in this retrospective, observational study. Of those patients, 34 underwent LVZ ablation of the LAAW after PVI. The LVZ was defined as that with a bipolar voltage of less than 0.5 mV. Ten (29.4%) patients who underwent LVZ ablation developed BiAT. One patient was excluded due to an inadequate evaluation of the P waves. Therefore, the final analysis included nine patients. We evaluated the precise circuits in both the LA and RA using a high‐density mapping system and analyzed the optimal treatment strategy and long‐term results. Informed consent was obtained from each patient. The study was approved by our hospital's institutional review board and conforms to the provisions of the Declaration of Helsinki (as revised in Fortaleza, Brazil, October 2013).

### 2.2 Ablation procedure

Patients were administered an oral anticoagulant for at least 1 month prior to the ablation procedure. All antiarrhythmic drugs were discontinued for at least five half‐lives before the ablation procedure. In all patients, transesophageal echocardiography or enhanced computed tomography was performed prior to the procedure to detect an LA thrombus.

The patients were administered propofol for general anesthesia and placed in the supine position. A 7‐Fr catheter (Inquiry Luma‐Cath, Irvine Biomedical) was placed within the coronary sinus (CS) via the subclavian vein, then one transseptal puncture was created using intracardiac echocardiography guidance (CARTO SOUND STAR, Johnson & Johnson). Two transseptal sheaths (SL0, St. Jude Medical, Inc and Agilis, St. Jude Medical, Inc) were introduced into the LA via the right femoral vein. Electroanatomic navigation was performed using the CARTO3 system (Biosense Webster) and a 20‐electrode mapping catheter (PENTARAY; Biosense Webster) or the RHYTHMIA HDx™ mapping system (Boston Scientific) and a 64‐electrode mapping catheter (Orion multipolar basket catheter; Boston Scientific). A 3.5‐mm tip open‐irrigated ablation catheter (THERMOCOOL SMARTTOUCH® SF, Biosense Webster, and FlexAbility™ Sensor Enabled, Abbott Medical Japan LLC) was used for the ablation procedure. Radiofrequency (RF) energy delivery was set at 30‐45 W with a temperature limit of 45°C, and irrigation was set at 2‐8 mL/min. Systemic anticoagulation was achieved using a heparin bolus. The target activated clotting time was >300 s and the heparin bolus was administered every 10‐20 min as needed.

### 2.3 Selection of the ablation site

Previous studies have reported that BiAT can be treated with MI or BB ablation.^6^, ^7^, ^8^ The BB spans a wide area, and its distribution differs among patients.^9^, ^10^ Therefore, the range of the BB ablation is not clear and a method for BB ablation has not yet been reported. It can be assumed that the BB in some patients with BiAT may be damaged during previous LAAW ablation procedures. A previous study reported that a P wave duration >100 ms in the inferior leads is associated with interatrial conduction disturbances and an obstructed BB.^11^ Therefore, BB ablation is more effective than MI ablation in patients with a P wave duration >100 ms, because BB is already obstructed and we speculated that BB block line could succeed easily. We ablated any residual potentials that were obstructed during previous procedures in the area of the BB.

### 2.4 Diagnosis of BiAT

Activation maps of AT in both the RA and LA were created for all patients using the CARTO3 or RHYTHMIA HDx™ systems. BiAT was diagnosed when the AT activation map indicated a reentrant circuit involving both atria and the post‐pacing interval (PPI) and tachycardia cycle length (TCL) of the entrainment pacing of the circuit was <20 ms different.

### 2.5 Procedure endpoint and follow‐up

After the termination of the BiAT, we induced tachycardia via overdrive burst pacing. The minimum pacing cycle length was set at 200 ms, as permitted by the local effective refractory period of the atrial tissue. Successful termination of the BiAT was defined as the inability to induce tachyarrhythmia. Patients were followed up at 1, 3, 6, 12, 18, 24, 30, and 36 months after the ablation procedure. During the follow‐up visits, a 12‐lead electrocardiogram (ECG) and 24‐hour Holter monitoring were performed. The recurrence of AF or AT was defined as an episode of either lasting more than 30 s.

### 2.6 Statistical analyses

Continuous variables were described as mean values and compared using the Mann‐Whitney U‐test. All analyses were conducted using Statistical Package for the Social Sciences (SPSS) version 18.0J software (SPSS Inc, IBM). Statistical significance was set at *P* < .05.

## 3 RESULTS

**FIGURE 1:**
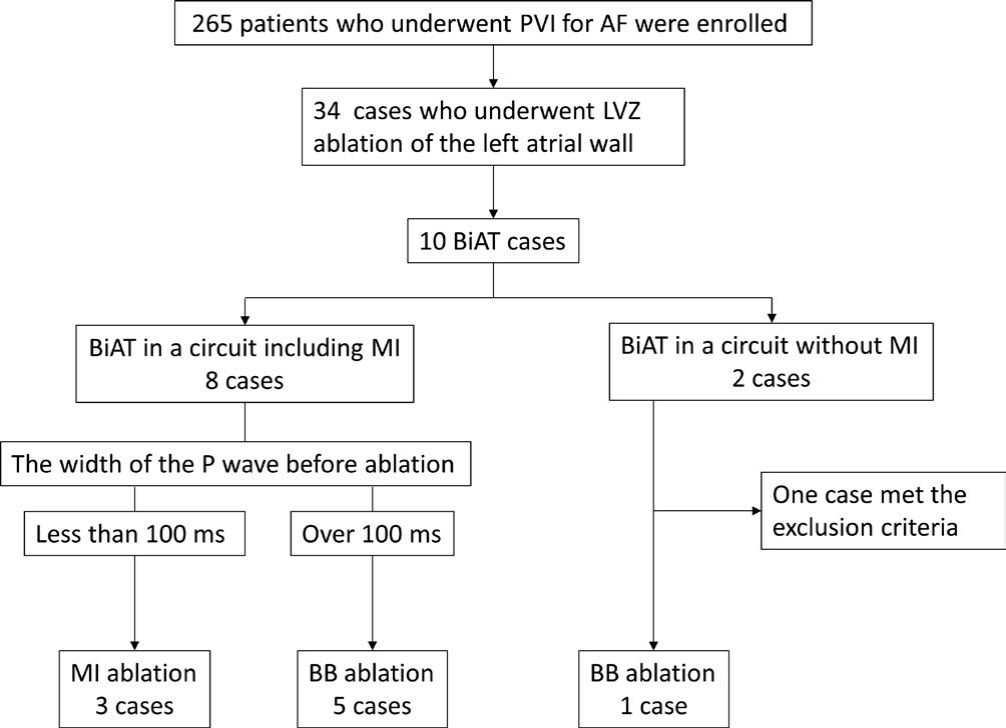
Patient flow chart

Thirty‐four patients who underwent LVZ ablation of the LAAW were enrolled in this study including 10 who developed BiAT (Figure 1). The BiAT propagated from the MI in eight patients. MI ablation was performed in three patients with BiAT from the MI and a P wave duration <100 ms BB ablation at the LA was performed in five patients with BiAT from the MI and a P wave duration >100 ms The P wave duration was measured using the final sinus rhythm before the recurrence of BiAT in the inferior lead. Two patients had BiAT without the involvement of the MI, including one with an implanted pacemaker due to sick sinus syndrome in whom the P waves could not be adequately evaluated before and after ablation; therefore, this patient was excluded from the final analysis.

### 3.1 Patient characteristics

The baseline characteristics of patients with BiAT who underwent MI or BB ablation are shown in Table 1. All but one patient (patient 8) had an enlarged LA diameter. Five patients (55%) had paroxysmal AF and 4 (44%) had persistent AF. Three patients (33%) had a history of peri‐mitral flutter (PMF). Overall, 3 patients underwent MI ablation (MI ablation group) and 6 underwent BB ablation (BB ablation group).

**TABLE 1:** Patient characteristics

Case	Ablation site	Age	Sex	AF type	CHA_2_DS_2‐_VASc	SHD	AADs	LVEF (%)	LAD (mm)	PMF	Prior MI ablation	Prior ablation
Case 1	MI	81	F	PAF	4	‐	bepridil	74	42.1	‐	‐	PVI, CTI, roofline, bottom line, anterior line
Case 2	MI	66	M	PeAF	2	‐	pilsicainide, bepridil	77.5	44.4	‐	‐	PVI, CTI, roofline
Case 3	MI	73	M	PeAF	2	Maze	‐	35.7	41.9	+	+	PVI, roofline
Case 4	BB	68	M	PAF	1	‐	‐	68	42.4	‐	‐	PVI
Case 5	BB	78	F	PAF	4	‐	bepridil, aprindine	52.3	45.7	+	‐	PVI, roofline, bottom line, anterior line
Case 6	BB	68	F	PAF	2	‐	cibenzoline	64	48	‐	‐	PVI, CTI, roofline, bottom line, anterior line
Case 7	BB	72	M	PeAF	3	‐	bepridil	66.4	45.1	+	‐	PVI, CTI, anterior line
Case 8	BB	69	F	PAF	2	‐	‐	69	35.7	‐	‐	PVI, roofline, bottom line, anterior line
Case 9	BB	77	F	PeAF	4	MVP, TAP	‐	42.8	40.2	‐	‐	CTI

Abbreviations: AADs, antiarrhythmic drugs; AF, atrial fibrillation; BB, Bachmann's bundle; BiAT, bi‐atrial tachycardia; CTI, cavotricuspid isthmus line ablation; LAD, left atrial dimension; LVEF, left ventricular ejection fraction; Maze, Maze procedure; MI, mitral isthmus; MVP, mitral valve plasty; PAF, paroxysmal atrial fibrillation; PeAF, persistent atrial fibrillation; PMF, peri‐mitral atrial flutter; PMI, pacemaker implantation; PVI, pulmonary vein isolation; SHD, structural heart disease; TAP, tricuspid annuloplasty; TCL, tachycardia cycle length.

In the MI ablation group, patient 1 had a history of ablation at the PVI, CTI, roofline, bottom line, and anterior line; patient 2 had a history of ablation at the PVI, CTI, and roofline; and patient 3 had a history of ablation at the PVI and roofline. Patient 3 had PMF and had undergone MI ablation in the past. The previous MI ablation was incomplete, leading to the development of BiAT during this study period. All three patients in the MI ablation group underwent roof linear ablation.

In the BB ablation group, patient 4 had had a history of ablation at the PVI; patients 5 and 8 both had a history of ablation at the PVI, roofline, bottom line, and anterior line; patient 6 had a history of ablation at the PVI, CTI, roofline, bottom line, and anterior line; and patient 7 had a history of ablation at the PVI, CTI, and anterior line. The tachycardia circuit in 5 patients (83%) who underwent BB ablation involved the MI. Two patients (33%; patients 5 and 7) had PMF and history of anterior linear ablation due to the LVZ involving much of the LAAW (Table 1).

### 3.2 Characteristics of BiAT

The median TCL of the BiAT was 270 ms in the MI ablation group and 350 ms in the BB ablation group (interquartile ranges (IQRs): 245‐360 ms and 270‐450 ms, respectively). Two patients in each group had a clockwise activation pattern and the remaining patients had counterclockwise activation patterns.

In the MI ablation group during this session, two patients (66%) underwent MI linear ablation at the endocardium only. MI ablation was performed at the endocardial and epicardial sides (via the CS) in the third patient in this group. The bi‐directional MI line block was confirmed by the differential site pacing from the CS. In the BB ablation group, all six patients underwent BB ablation from the LA side.

There were no significant differences between the two groups in the ablation time required for the termination of the BiAT (MI ablation: 284 s, IQR: 187‐782 s; BB ablation: 216 s, IQR: 28.8‐522 s; *P* = .71) or the total ablation time (MI ablation: 676 s, IQR: 427‐1167 s; BB ablation: 490 s, IQR: 233‐919 s; *P* = .38) (Table 2).

**TABLE 2:** Characteristics of nine patients with bi‐atrial tachycardia

Case	Ablation site	System	TCL (ms)	Direction of Propagation	Ablation Site	Time to termination (sec)	Total ablation time (sec)	Pre P wave (msec)	Post P wave (msec)	Change of the P wave duration (msec)	Recurrence	Follow up term (month)
Case 1	MI	Rhythmia	270	CW		782	1167	97	102	5	‐	24
Case 2	MI	Rhythmia	450	CCW		187	427	95	95	0	‐	18
Case 3	MI	CARTO	220	CW		284	676	91	97	6	‐	36
Case 4	BB	Rhythmia	450	CCW	LA	37	210	101	134	33	‐	22
Case 5	BB	CARTO	360	CCW	LA	4	361	100	106	6	+	36
Case 6	BB	CARTO	340	CW	LA	420	882	100	144	44	‐	33
Case 7	BB	Rhythmia	470	CCW	LA	382	618	234	343	109	‐	32
Case 8	BB	CARTO	250	CCW	LA	828	1028	100	120	20	‐	32
Case 9	BB	Rhythmia	270	CW	LA	240	49	103	150	47	‐	27

Abbreviations: BB, Bachmann's bundle; CCW, counterclockwise; CW, clockwise; LA, left atrium; MI, mitral isthmus.

### 3.3 Representative cases of BiAT

#### 3.3.1 MI ablation in a patient with BiAT involving the MI and a P wave duration <100 ms

**FIGURE 2:**
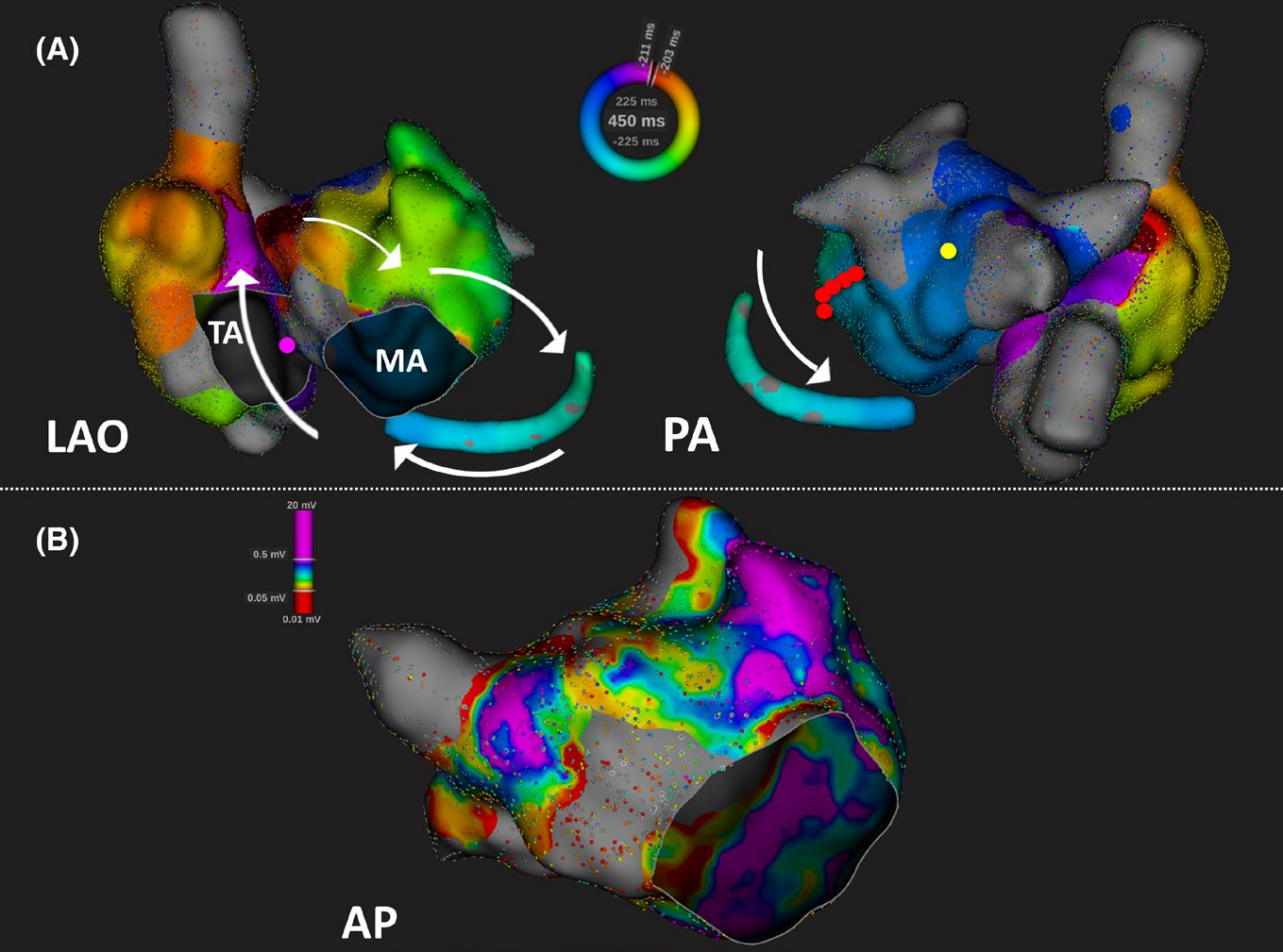
Activation and voltage map of bi‐atrial tachycardia (BiAT) in patient 2, created using the Rhythmia mapping system. (A) Patient 2 was found to have a clockwise activation pattern of bi‐atrial tachycardia (BiAT). The circuit extends from the mitral isthmus to the right atrial septum via the coronary sinus. The post‐pacing intervals are less than the tachycardia cycle length (TCL) by 20 ms from the RA septum (pink tag) and are more than TCL by 20 ms from the left atrium posterior wall (yellow tag). The BiAT was terminated via mitral isthmus linear ablation (red tags). (B) The voltage map indicates that the low voltage zone is wide in the left atrial anterior wall. The low voltage zone is defined as that under 0.05 mV

The activation map created using the RHYTHMIA HDx™ system indicated a tachycardia circuit in the CS of the RA septum via the MI with a clockwise rotation that returned to the LA via the BB in patient 2 (Figure 2A, B). The PPI was equal to the TCL at the RA septum. MI ablation performed as the P wave duration during sinus rhythm was 95 ms The BiAT was successfully terminated using MI ablation.

#### 3.3.2 BB ablation in a patient with BiAT including the MI and a P wave duration >100 ms

**FIGURE 3:**
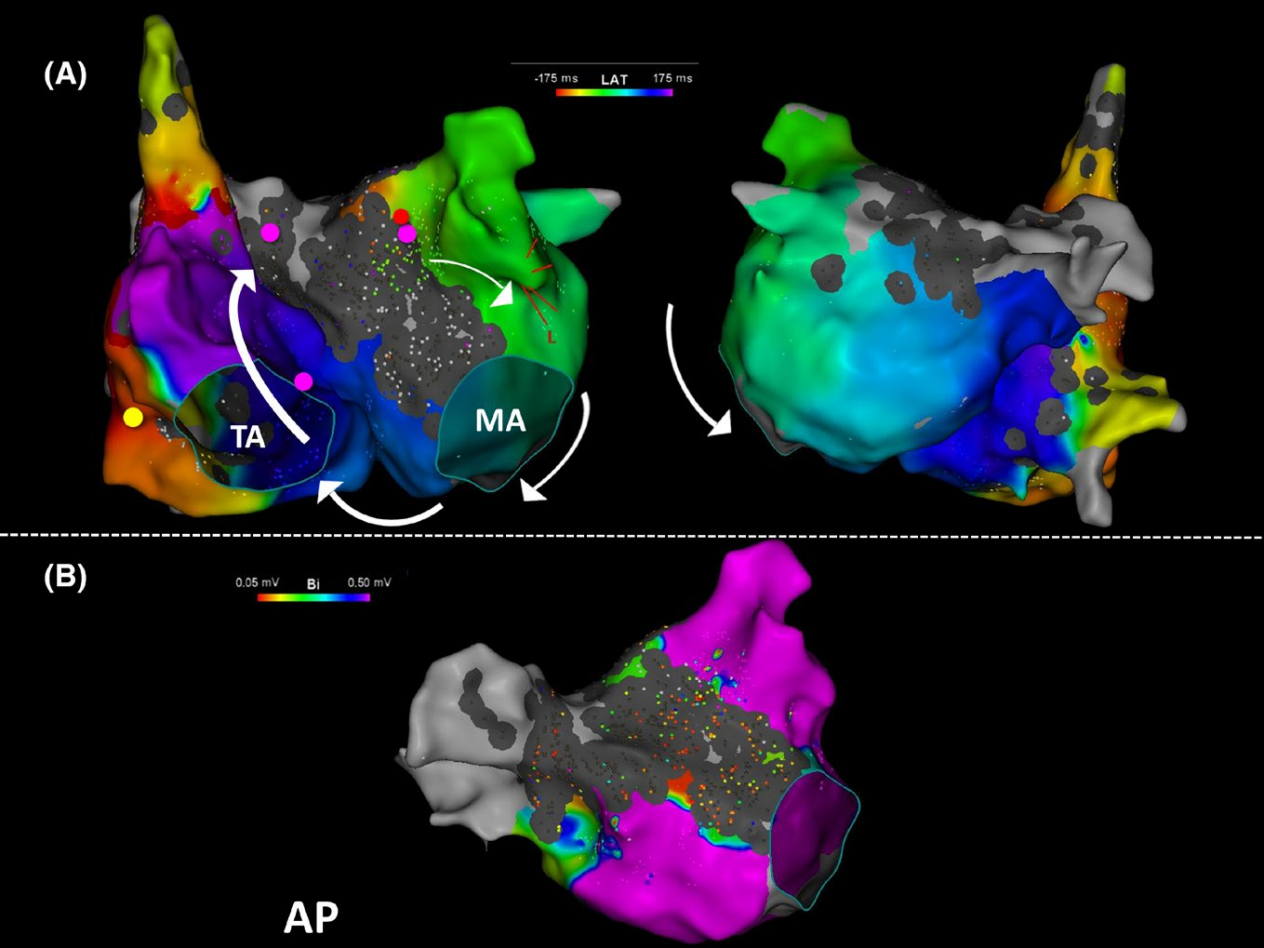
Activation and voltage maps of bi‐atrial tachycardia in patient 6 created using the CARTO3 mapping system. (A) Patient 6 was found to have a clockwise activation pattern of bi‐atrial tachycardia (BiAT). The tachycardia circuit includes the left atria through Bachmann's bundle (BB) and the right atria (RA) intermediary of the mitral isthmus and coronary sinus (CS). Although the lateral RA is the earliest site, the post‐pacing intervals (PPI) do not correspond with the tachycardia cycle length (yellow tag). The PPIs of the BB, distal CS, and RA septum are less than the TCL by 20 ms (pink tags). The BiAT was terminated via BB ablation (red tags). (B) The voltage map indicates that the low voltage zone is wide in the left atrial anterior wall

The activation map created using the CARTO3 system indicated that the wavefront of the tachycardia circuit propagated from the LA and passed through the MI and CS into the BB on the RA side with a clockwise rotation in patient 6 (Figure 3A, B). Although the lateral RA may have been the earliest activation site, this was not indicated by the PPI‐TCL interval. The PPI‐TCL interval revealed the proximity of the reentrant circuit to the BB and RA septum. As the P wave duration during sinus rhythm was 100 ms, BB ablation was performed.

#### 3.3.3 BB ablation in a patient with BiAT not including the MI and a P wave duration >100 ms

**FIGURE 4:**
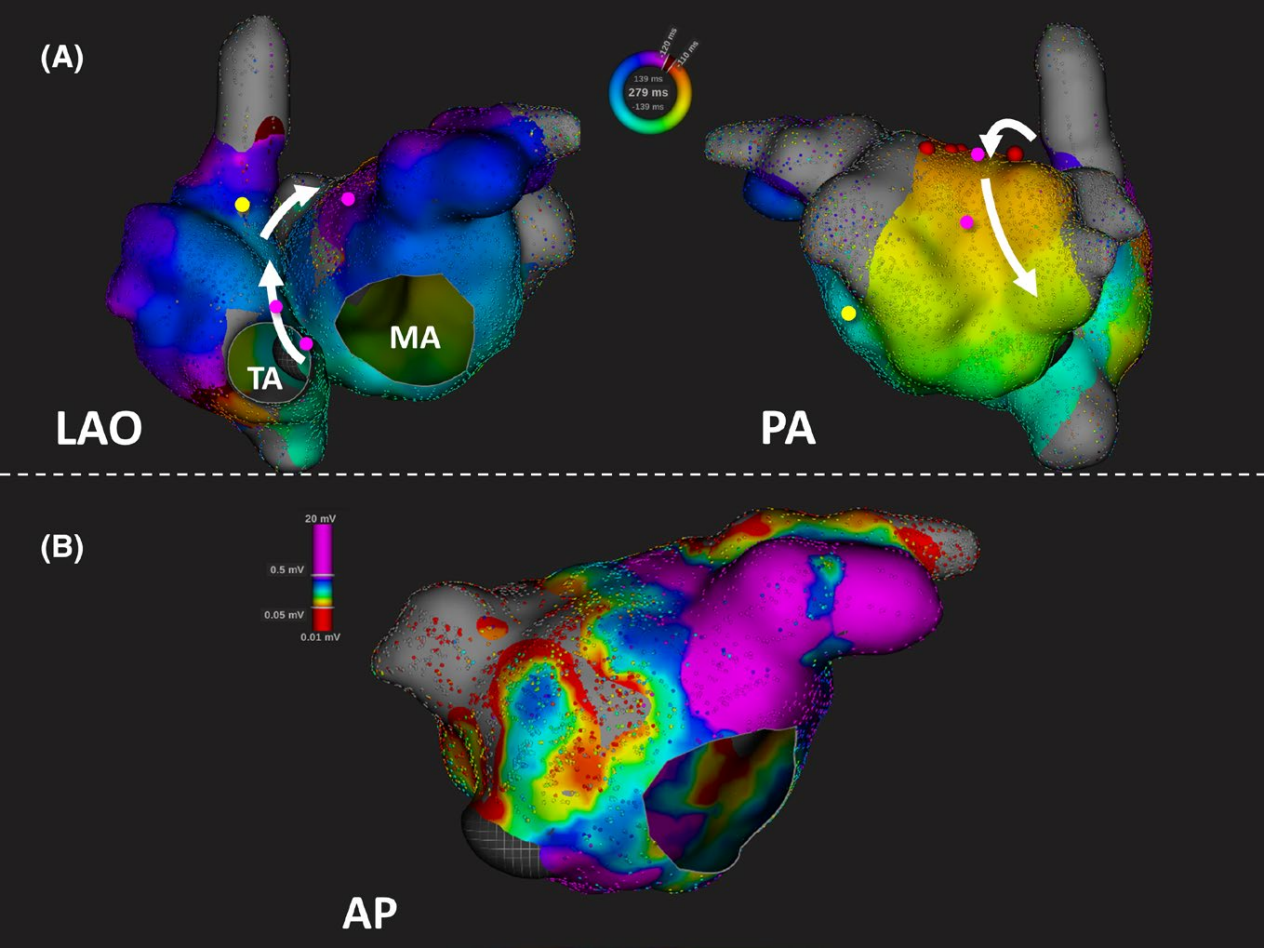
Activation and voltage maps of bi‐atrial tachycardia in patient 9 created using the Rhythmia mapping system. (A) Patient 9 was found to have a clockwise activation pattern of bi‐atrial tachycardia (BiAT). The tachycardia circuit is transmitted from Bachmann's bundle (BB) to the left atrium to the right atrium (RA) via the septum. The post‐pacing interval (PPI) from the roof and RA septum is equal to the tachycardia cycle length (TCL) (pink tags). The PPIs do not correspond with the mitral isthmus (yellow tag). The BiAT was terminated via BB ablation (red tags). (B) The voltage map indicates that the low voltage zone is wide in the left atrial anterior wall

The activation map created using the RHYTHMIA HDx™ system showed that the wavefront of the tachycardia circuit propagated from the posterior wall of the LA to the RA septum returned to the LA via the BB with a clockwise rotation, and did not involve the MI in patient 9 (Figure 4A, B). The PPI was equal to the TCL at the RA septum and the roof of the LA. Moreover, the PPI of the posterior wall of the LA was less than the TCL by 20 ms The P wave duration was 103 ms during sinus rhythm before ablation; therefore, BB ablation, including the roof of the LA, was performed. The BB ablation successfully terminated the BiAT.

### 3.4 P wave characteristics after the ablation procedure

In the MI ablation group, the P wave duration after ablation was slightly prolonged compared to that before ablation in two patients (66%). The P wave duration after ablation was prolonged compared to that before ablation in six patients (100%) in the BB ablation group. The prolongation of the P wave duration was significantly greater in the BB ablation group (38.5 ms, IQR: 16.5‐62.5 ms) than in the MI ablation group (5 ms, IQR: 0‐6.0 ms) (*P* = .024; Table 2).

**FIGURE 5:**
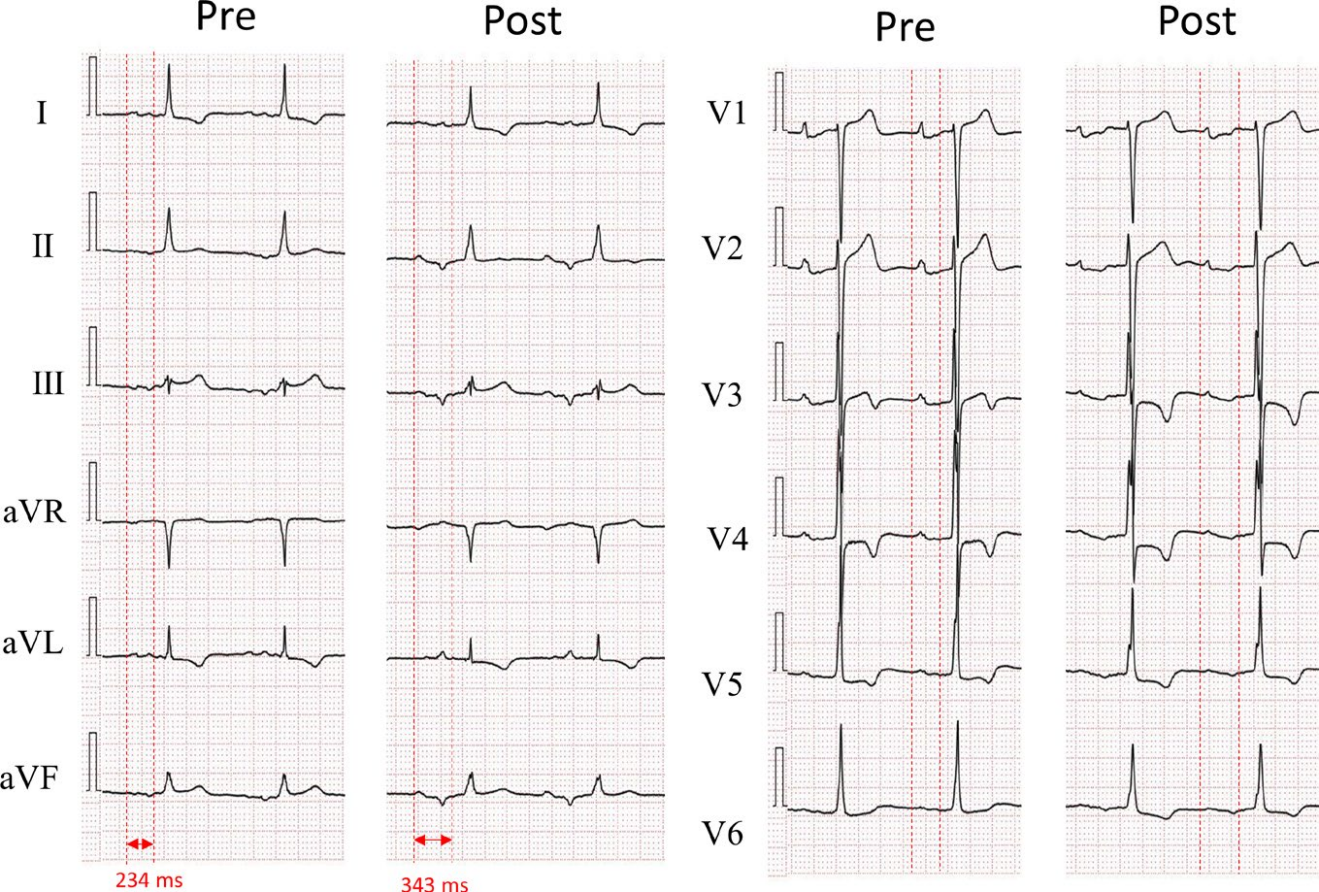
Changes in P wave duration and morphology before and after ablation. In patient 7, the P wave morphology changed to an obvious biphasic wave in the inferior leads of a 12‐lead electrocardiogram and its duration is prolonged from to 343 ms after ablation from 234 ms before ablation

The P wave duration was prolonged from 234 ms to 343 ms in patient 7 who underwent BB ablation. The P wave morphology showed a prolonged, biphasic P wave in the inferior leads on ECG (Figure 5).

### 3.5 Follow‐up

The mean follow‐up period was 26.9 months (IQR: 18‐36 months). AT recurrence occurred in one patient (11%) 11 months after the ablation procedure (Table 2). The P wave duration in this patient was not prolonged by >20 ms and was <120 ms This patient did not undergo a repeat catheter ablation and continued to receive antiarrhythmic drugs.

## 4 DISCUSSION

We compared the outcomes of ablation at the MI or BB in patients with BiAT and found that the change in the P wave duration was significantly greater in patients who underwent BB ablation than in patients who underwent MI ablation. We also found no significant difference in the ablation times between the two groups. The recurrence rate over a 26.9‐month follow‐up period was 11%.

Previous studies have reported that BiAT can be terminated by BB ablation.^4^, ^6^, ^7^, ^8^ The BB is a muscle bundle that connects the RA and LA, spanning from the RA appendage to the LA appendage, requiring a large area of ablation.^9^, ^10^ As ablation from the endocardium at the BB is more difficult than ablation at the MI, we predicted that the ablation time in the BB group would be longer than that in the MI group. However, there was no significant difference in the ablation time between the two groups. Bayes et al reported that P wave durations over 100 ms suggest impairment of the interatrial conduction, including conduction through the BB.^9^ In this study, it is considered that the BB ablation time was reduced because BB ablation was performed only for the residual potential in patients in whom the BB was already obstructed due to perform past procedures.

We believed that the BB would be damaged during the previous LAAW ablation that the patients in this study underwent.^11^, ^12^ A previous study reported that the morphology of the P wave can be used to identify LA breakthrough sites (such as the BB, fossa ovalis, or CS) and the corresponding atrial propagation site.^13^ Impaired interatrial conduction may be seen as the prolongation of the P wave on a 12‐lead ECG due to the conduction disturbance along the BB. In addition, advanced interatrial block caused by the obstruction of the BB may be seen as a prolonged, biphasic P wave in the inferior leads on ECG. Bayes et al also reported that the prolonged (≧120 ms) monophasic and biphasic P waves on ECG indicate interatrial blocks such as the obstructed BB.^11^

The P wave duration was prolonged by >20 ms and was 120 ms or more after the ablation in five patients in the BB ablation group. In patient 7, the P wave duration was >120 ms prior to the ablation due to the conduction disturbance caused by past procedures. The P wave morphology of patient 7 changed to a biphasic wave that remained prolonged, as indicated by an advanced interatrial block (Figure 5). Patient 5 had a prolonged P wave duration of 106 ms after ablation, which was insufficiently prolonged. We believe that BB ablation might have been insufficient in this patient, leading to a recurrence, as the P wave duration was not prolonged by <20 ms and was <120 ms Determining the endpoint of the BB ablation was challenging as the BB covers a wide area leading from the RA to the LA and the ablation points were unclear. Therefore, the prolongation of the P wave duration by >20 ms and a duration ≥120 ms after ablation was used to determine the success of the BB ablation procedure. One possible complication of the BB ablation procedure is a worsened interatrial conduction disturbance, causing heart failure. In this study, no patients developed heart failure after BB ablation.

The mean follow‐up period in this study was 26.9 months. To our knowledge, this is the longest follow‐up period reported for patients with BiAT. One patient (11%) experienced recurrent BiAT after the ablation procedure, which is consistent with a previous study that reported a recurrence rate of BiAT after ablation of 12.5%.^5^

## 5 LIMITATIONS

This study is not without limitations, which include the retrospective design and the small number of patients. Larger studies are necessary to confirm the results of this study. In this study, the PPI at the RA septum was measured when BiAT was diagnosed; however, this pacing may have also been present on the contralateral side (LA septum side). PPI at the LA septum side was not measured in all patients due to the wide LVZ in some patients that prevented the pacing at the LA septum from being captured. Therefore, the inclusion of the LA septal side in the circuit was not confirmed in all patients. In addition, the possibility of a bi‐directional block at the BB site was not evaluated in all patients who underwent BB ablation, though two activation maps from different pacing sites were created to confirm a bi‐directional block in one patient. We previously reported that activation maps of the RA during LA appendage pacing or those of the LA during sinus rhythm reveal that the earliest activation sites were the CS and the posterior LA. These activation maps indicated that there were no interatrial conductions through the BB.^8^ Finally, in this study, we did not evaluate LA function before or after the procedure. Previous studies have reported that increased LA volume and diameter indicate LA dysfunction and are related to the risk of the development of AF and recurrent AF.^14^, ^15^ However, another previous study reported that LA ejection fraction (LAEF) is a more sensitive marker of early LA remodeling than LA enlargement.^16^ Impairment of LAEF precedes LA dilatation in patients with paroxysmal AF, and early LA remodeling can be detected by a decrease in LAEF without LA enlargement.^16^

Moreover, LAEF dysfunction after ablation in patients with AF has been reported as a risk factor for the recurrence of AF.^17^ In this study, the LA diameter before and after ablation was evaluated. All patients in this study had a history of ablation procedures. The LA diameter increased in all patients, except for patient 8. However, in this study, we did not measure LA volume and cannot evaluate LAEF or LA function.

## 6 CONCLUSIONS

Ablation of either the MI or BB would be an effective strategy to treat patients with BiAT in which baseline duration of the P wave may suggest it as a proper choice. Both P wave prolongation by >20 ms and a P wave duration of ≥120 ms after BB ablation may an indicative of a successful BB ablation for BiAT.

## CONFLICT OF INTEREST

Authors declare no conflict of interests for this article.

## Supporting information

Supplementary Material

Supplementary Material

Supplementary Material
